# Rooting for order: How CIKs keep lateral growth in check

**DOI:** 10.1093/plphys/kiae621

**Published:** 2024-11-21

**Authors:** Alicja B Kunkowska, Nicola Trozzi

**Affiliations:** Assistant Features Editor, Plant Physiology, American Society of Plant Biologists; PlantLab, Institute of Plant Sciences, Sant’Anna School of Advanced Studies, 56010 Pisa, Italy; Assistant Features Editor, Plant Physiology, American Society of Plant Biologists; Department of Computational and Systems Biology, John Innes Centre, Norwich Research Park, Norwich NR4 7UH, UK; Department of Plant Molecular Biology, University of Lausanne, CH-1015 Lausanne, Switzerland

Roots are essential for plant survival, anchoring them in the soil and absorbing water and nutrients they rely on to grow. The ability of a plant to thrive in different environments is influenced by the efficiency of its root system, which is shaped by the formation and arrangement of lateral roots branching from the main root (Gruber et al. 2013; Bao et al. 2014). Proper lateral root development maximizes soil exploration, optimizes resource acquisition, and helps plants adapt to various environmental conditions (Kenrick and Strullu-Derrien 2014; Shekhar et al. 2019).

RECEPTOR-LIKE KINASE 7 (RLK7) plays a key role in regulating lateral root patterning by detecting inhibitory signals from the TARGET OF LBD SIXTEEN 2 (TOLS2) peptide, which is secreted by neighboring cells. TOLS2 triggers the upregulation of the APETALA2-type transcription factor PUCHI, downstream of RLK7 (Hirota et al. 2007; Toyokura et al. 2019). This pathway limits lateral root initiation, ensuring even spacing of lateral roots and preventing resource competition in the soil. However, the molecular mechanisms by which RLK7 perceives and transduces the TOLS2 signal remain unsolved.

In this issue of *Plant Physiology*, Meng et al. (2024) identify a novel role for CLAVATA3 INSENSITIVE RECEPTOR KINASES (CIKs) in the RLK7-mediated signaling pathway. The authors propose that RLK7 works together with CIK proteins to transduce the TOLS2 signal during lateral root development (Fig. 1). Genotypic and phenotypic analyses revealed that *CIKs* are expressed similarly to *RLK7* in lateral root founder cells and lateral root primordia (LRP). Moreover, the *cik1/2/4/5/6* mutants displayed a phenotype similar to that of *rlk7-3* (Toyokura et al. 2019), with increased LRP and lateral roots. These findings suggest that CIKs and RLK7 are likely in the same pathway to regulate LRP initiation and spacing.

**Figure 1.**
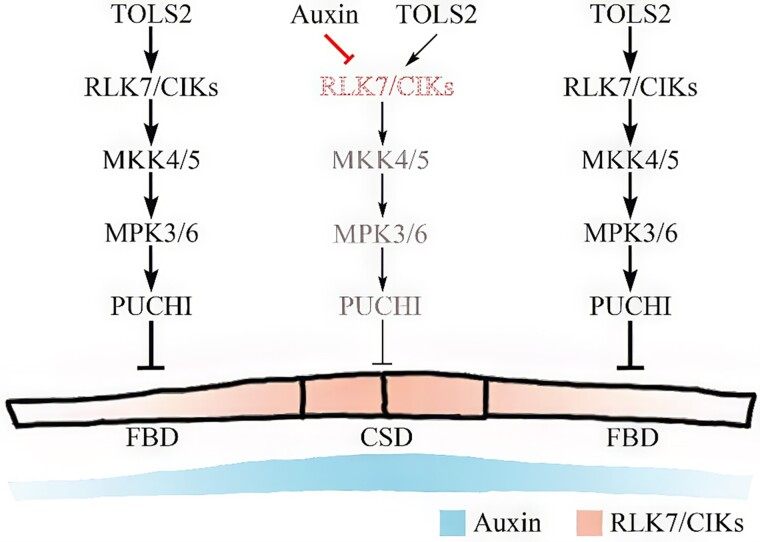
CIKs regulate the initiation and spacing of LRP via auxin and TOLS2 signaling. During lateral root initiation, high auxin levels in central smaller daughter (CSD) cells direct CIK proteins to the vacuole, disrupting TOLS2 signaling and promoting increased cell division. In contrast, flanking bigger daughter (FBD) cells, which have lower auxin concentrations, maintain active TOLS2 signaling, inhibiting further root initiation. Thin black arrows and brown text illustrate weakened signaling in auxin-enriched regions, while red lines and dotted text represent the promotion of vacuolar localization of RLK7/CIK complexes by auxin. Adapted from Meng et al. (2024).

Co-immunoprecipitation assays revealed interactions between RLK7 and the CIK proteins (CIK1, CIK2, CIK3, and CIK5), supporting the idea that CIKs are integral to the RLK7 signaling complex. The interaction between RLK7 and CIK4 was confirmed using yeast 2-hybrid and bimolecular fluorescence complementation assays. These results suggest that CIK proteins form part of a receptor complex with RLK7, which mediates the perception and transduction of the TOLS2 signal.

In wild-type plants, TOLS2 treatment reduced LRP number, consistent with the role of RLK7s in suppressing LRP initiation. However, TOLS2 treatment had no effect on *rlk7-3* or *cik1/2/4/5/6* mutants, highlighting the necessity of both RLK7 and CIKs in TOLS2 signaling. Additionally, TOLS2 treatment enhanced CIK phosphorylation in wild-type plants but not in *rlk7-3* mutants, suggesting that CIK phosphorylation is also integral to the signaling pathway. Moreover, *PUCHI* expression was unresponsive to TOLS2 treatment in *cik1/2/4/5/6* mutants, confirming that CIKs are essential for transmitting the TOLS2 signal and activating downstream responses.

The study also establishes a connection between CIK proteins and the MKK4/5-MPK3/6 cascade, which, among other functions, is a well-known component of auxin signaling in RLK pathways (Jourquin et al. 2022). In wild-type plants, TOLS2 treatment enhances MPK3/6 phosphorylation, but this response is absent in both *cik1/2/4/5/6* and *rlk7-3* mutants, suggesting that CIKs act upstream of MPK3/6 in regulating lateral root initiation (Fig. 1). These results indicate potential cross-talk between the TOLS2-RLK7 pathway and auxin-mediated signaling, which could reveal new layers of regulation in lateral root development.

Meng et al. (2024) provide compelling evidence that CIK proteins, previously associated with shoot development, also play a critical role in shaping root system architecture by regulating lateral root spacing in Arabidopsis. By identifying CIKs as key regulators of lateral root development, this study opens new opportunities to improve crop performance through targeted manipulation of root architecture. Future research could explore how environmental factors, such as nutrient availability or drought, affect the CIK-RLK7 signaling pathway to better understand the adaptability of root systems to changing conditions.

## Data Availability

No data were generated or analysed in this study.

## References

[kiae621-B1] Bao Y , Aggarwal P, Robbins NE 2nd, Sturrock CJ, Thompson MC, Tan HQ, Tham C, Duan L, Rodriguez PL, Vernoux T, et al Plant roots use a patterning mechanism to position lateral root branches toward available water. Proc Natl Acad Sci U S A. 2014:111(25):9319–9324. 10.1073/pnas.1400966111 24927545 PMC4078807

[kiae621-B2] Gruber BD , Giehl RF, Friedel S, von Wirén N. Plasticity of the Arabidopsis root system under nutrient deficiencies. Plant Physiol. 2013:163(1):161–179. 10.1104/pp.113.218453 23852440 PMC3762638

[kiae621-B3] Hirota A , Kato T, Fukaki H, Aida M, Tasaka M. The auxin-regulated AP2/EREBP gene PUCHI is required for morphogenesis in the early lateral root primordium of Arabidopsis. Plant Cell. 2007:19(7):2156–2168. 10.1105/tpc.107.050674 17630277 PMC1955702

[kiae621-B4] Jourquin J , Fernandez AI, Parizot B, Xu K, Grunewald W, Mamiya A, Fukaki H, Beeckman T. Two phylogenetically unrelated peptide-receptor modules jointly regulate lateral root initiation via a partially shared signaling pathway in Arabidopsis thaliana. New Phytol. 2022:233(4):1780–1796. 10.1111/nph.17919 34913488 PMC9302118

[kiae621-B5] Kenrick P , Strullu-Derrien C. The origin and early evolution of roots. Plant Physiol. 2014:166(2):570–580. 10.1104/pp.114.244517 25187527 PMC4213089

[kiae621-B6] Meng X , Ye R, Cao J, Tao L, Wang Z, Kong T, Hu C, Yi J, Gou X. CLAVATA3 INSENSITIVE RECEPTOR KINASEs regulate lateral root initiation and spacing in Arabidopsis. Plant Physiol. 2024. 10.1093/plphys/kiae540 39387495

[kiae621-B7] Shekhar V , Stöckle D, Thellmann M, Vermeer JEM. The role of plant root systems in evolutionary adaptation. Curr Top Dev Biol 2019:131:55–80. 10.1016/bs.ctdb.2018.11.011 30612630

[kiae621-B8] Toyokura K , Goh T, Shinohara H, Shinoda A, Kondo Y, Okamoto Y, Uehara T, Fujimoto K, Okushima Y, Ikeyama Y, et al Lateral inhibition by a peptide hormone-receptor cascade during Arabidopsis lateral root founder cell formation. Dev Cell. 2019:48(1):64–75.e5. 10.1016/j.devcel.2018.11.031 30581155

